# Carbon loading in airway macrophages to traffic-derived particulate matter air pollution

**DOI:** 10.1186/2049-3258-73-S1-P19

**Published:** 2015-09-17

**Authors:** Yang Bai, Sven Seys, Tim Nawrot, Benoit Nemery

**Affiliations:** 1 KULeuven, Leuven, Belgium; 2 Hasselt University, Hasselt, Belgium

## Background and aims

Epidemiological studies indicate that the health outcomes of exposure to urban air pollution are driven by exposure to elemental carbon (EC) emitted from traffic-related fuel combustion. One possible method for assessing chronic exposure to EC in individual subjects consists of estimating the amount of carbonaceous particles present in alveolar macrophages (AM)s. However, a number of issues remain to be solved regarding this biomarker. We studied the reproducibility and the kinetics of this biomarker by making repeated measurements over one year in healthy subjects, with some of them having moved from a highly polluted area (PM_10_ > 30 μg/m^3^) to a moderately polluted area (PM_10_ 20-30 μg/m^3^).

## Methods

In this follow-up study, international students undergo a first assessment shortly after their arrival in Belgium, and then 7 further assessments every 6 weeks. For comparison, students having resided in Belgium for more than one year are examined concurrently. AM are obtained using induced sputum and the surface of carbon loading (m^2^) is measured in 50 AM by image analysis.

## Results

The preliminary data of this ongoing study indicate that the carbon loading in AM remains stable over the observation period. However, in subjects who moved from a highly polluted area (n=3, so far), a pronounced decrease in average carbon loading (2.86 to 1.33) is observed between their first second visits.

## Conclusions

The stability of the carbon loading of AM in induced sputum over a period of several months suggests that this biomarker is suitable to estimate chronic exposure to traffic-related air pollution in individuals. This is confirmed by the observation of a sharp decline in carbon loading in AM over a period of six weeks when moving from an area of high pollution to an area of moderate pollution.

